# Hydrogeogenic fluoride in groundwater and dental fluorosis in Thai agrarian communities: a prevalence survey and case–control study

**DOI:** 10.1186/s12903-021-01902-8

**Published:** 2021-10-22

**Authors:** Chanapong Rojanaworarit, Luz Claudio, Nopporn Howteerakul, Auamduan Siramahamongkol, Pattraravith Ngernthong, Pornpimol Kongtip, Susan Woskie

**Affiliations:** 1 Department of Health Professions, School of Health Professions and Human Services, Hofstra University, Hempstead, NY USA; 2 Department of Environmental Medicine and Public Health, Icahn School of Medicine at Mount Sinai, New York, NY USA; 3 Department of Epidemiology, Faculty of Public Health, Mahidol University, Bangkok, Thailand; 4 Nakhon Pathom Provincial Public Health Office, Nakhon Pathom, Thailand; 5 Department of Occupational Health and Safety, Faculty of Public Health, Mahidol University, Bangkok, Thailand; 6 Department of Public Health, University of Massachusetts Lowell, Lowell, MA USA

**Keywords:** Dental fluorosis, Groundwater, Oral health, Environmental health, Epidemiology

## Abstract

**Background:**

Dental fluorosis can be a disease of social inequity in access to safe drinking water. This dental public health issue becomes prominent in socially disadvantaged agrarian communities in fluoride endemic areas where the standard irrigation system is unavailable and groundwater containing natural fluoride is the major drinking water source. This study aimed to determine the prevalence and severity of dental fluorosis in children and to evaluate its association with fluoride in groundwater in the aforementioned setting in Thailand.

**Methods:**

A cross-sectional survey of 289 children in Nakhon Pathom Province was conducted in 2015. Children with very mild to severe fluorosis were regarded as ‘cases’ while their counterparts were ‘controls’ for a subsequent case–control study. Records of fluoride concentrations in groundwater used for household supply corresponding to resident and number of years by age of each child during 2008–2015 were retrieved. Other exposure variables were measured using a questionnaire. Prevalence ratio (PR), a measure indicating the relative effect of different levels of fluoride on dental fluorosis, was obtained from Poisson regression with robust standard error.

**Result:**

There were 157 children with very mild to moderate dental fluorosis (54.3% prevalence). The univariable analysis revealed that the prevalence of dental fluorosis among children with fluoride concentrations in water sources of 0.7–1.49 (index category 1) and ≥ 1.5 ppm (index category 2) was 1.62 (95% CI; 0.78, 3.34) and 2.75 (95% CI; 1.42, 5.31) times the prevalence among those with fluoride < 0.7 ppm (referent category). After adjusting for all covariates, the adjusted prevalence ratios in both index categories were 1.64 (95% CI; 0.24, 11.24) and 2.85 (95% CI; 0.44, 18.52) which were close to their corresponding crude estimates. Since the magnitude of confounding, measured by (PR_crude_–PR_adjusted_)/PR_adjusted_, were less than 10% for both index categories; this indicated the limited confounding effect of all covariates.

**Conclusions:**

In fluoride endemic areas, groundwater containing natural fluoride utilized for household consumption resulted in high dental fluorosis prevalence, particularly in the groundwater with fluoride concentrations of ≥ 1.5 ppm.

**Supplementary Information:**

The online version contains supplementary material available at 10.1186/s12903-021-01902-8.

## Background

Etiologic patterns of dental fluorosis vary across different populations and settings. To explain the variation in the etiologic mechanisms of oral diseases, epidemiological models including the epidemiologic triad and Sufficient-Component Cause Model have been previously applied [1–3].

A classical model of the epidemiologic triad considers the interrelationship between host, agent, and environment in the causation of diseases [4]. In fluoride non-endemic areas, the environmental influence in the etiology of dental fluorosis is limited because the exposure of the susceptible population–children between the age of 6–8 years old with developing dental enamel [5]—is limited to manufactured fluoride products—i.e., fluoride toothpaste [6]. In contrast, the likelihood of host-agent interaction is heightened in fluoride endemic areas. Since the groundwater can serve as an abundant source of natural environmental fluoride, the risk of exposure to excessive fluoride is increased, as is the potential for dental fluorosis [7, 8]. Nonetheless, simply residing in a geological fluoride belt is not sufficient to cause dental fluorosis. Multiple factors are required to promote that necessary host-agent interaction. In a unique setting of socially disadvantaged rural agrarian communities, social determinants of health, including the low socioeconomic status and social inequity in access to a safe drinking water source, can increase the likelihood of excessive environmental fluoride exposure through the use of natural water sources [9].

Since this etiologic pattern requires not only the geographical presence of environmental fluoride but also other causal factors, the Sufficient-Component Cause Model can be applied to further elaborate the causal mechanism [10]. A sufficient cause in the model refers to a minimum set of causal factors or ‘component causes’ that inevitably produce disease [10, 11]. In the fluoride endemic areas, there may be several sufficient causes of dental fluorosis that vary in their components, although some components may be shared among various sufficient causes [12]. A sufficient cause that produces dental fluorosis in one individual may consist of three component causes comprising: (1) the presence of high fluoride in environmental sources, (2) lack of safe drinking water sources, and (3) low socioeconomic status that drives hosts to use the natural sources of water [13]. While a sufficient cause in another individual may comprise five-component causes including (1) swallowing of fluoride toothpaste, (2) use of local water sources for formula instead of breast milk, and (3–5) the three-component causes similar to those in the first individual [14, 15]. Nonetheless, dental fluorosis in individuals sharing the same sufficient cause components can still vary in severity levels. This suggests the need for a probabilistic causality approach [16] in dental fluorosis research to further describe the probability of an effect given a particular level of exposure [16]. Furthermore, causal directed acyclic graphs (DAGs) which is a graphic tool to visualize a causal structure can be applied to provide a better understanding of variables’ roles—i.e., exposure and confounder—in the etiology of dental fluorosis [17, 18].

In the environment, fluoride naturally occurs in rocks, soil, water, plants, animals, and human beings [8]. Fluoride-containing rocks such as fluorite (CaF_2_) and fluorapatite [Ca_5_(PO_4_)_3_F] release fluoride into soil and water through weathering process and dissolution in water and give rise to exposable environmental sources of fluoride [19]. Fluoride concentration in surface water such as rivers is normally lower than 0.1 mg per liter (mg/L) or parts per million (ppm) [20]. Nonetheless, fluoride concentration in the groundwater varies greatly and can be considerably higher, depending on composition in the host rock, climate, and hydrogeology [21]. Since fluoride in the human body is mainly obtained from drinking water, the inappropriate source of drinking water can result in excessive fluoride intake. The optimal fluoride concentration in drinking water is recommended not to exceed 0.7 mg/L [20, 22]. Excessive fluoride exposure during the development of dental organs would lead to the mineralization defect which causes dental fluorosis [5]. The critical period of dental fluorosis development in permanent dentition is from birth to 8 years old [23]. Therefore, dental fluorosis can be prevented by avoiding excessive fluoride intake in children age under 8 [24]. Investigation of natural water sources used for drinking and their association to the prevalence of dental fluorosis would provide evidence for the practical management of the natural sources of household water supply to control and prevent this disease in the community.

In the context of Thailand, there are large fluoride endemic areas mainly in the North and the West [21, 25]. In several villages of Bang Len, a district in Nakhon Pathom Province, Thailand, an excessive amount of fluoride is found in local drinking water sources and the common occurrence of dental fluorosis in children was observed by local public health professionals. Nonetheless, the magnitude and distribution of dental fluorosis corresponding to drinking water sources with varying fluoride concentrations have not been investigated. The provincial dental public health officers established a dental fluorosis surveillance system in these fluoride endemic areas to identify, control, and prevent dental fluorosis. The purposes of this study were to utilize this surveillance data to identify the prevalence and severity of dental fluorosis in children and to further evaluate a hypothesis that natural fluoride in the groundwater used for household water supply with concentration exceeding 0.7 ppm would increase the risk of dental fluorosis among children in these socially disadvantaged rural agrarian communities in Thailand.

## Methods

### Study design

This study comprises two phases of investigation: a cross-sectional survey to determine the prevalence and severity of dental fluorosis and a case–control study to examine the plausible association between environmental fluoride in groundwater used for household consumption and dental fluorosis occurrence. Application of two epidemiological designs in one study-to estimate the prevalence of disease by a cross-sectional survey before evaluation of the disease’s associated factors using case–control study-has previously been illustrated in several studies [26–28].

### Study setting and participants

The study was undertaken in Bang Len District, Nakhon Pathom Province, Thailand in 2015. Five subdistricts of Bang Len; including Bang Luang, Hin Mun, Bang Sai Pa, Sai Ngam, and Nin Phet; were selected from the total of 15 subdistricts due to the unique characteristics of (a) being fluoride endemic areas with pre-existing records of fluoride concentrations in the village water sources beyond 0.7 ppm, (b) being rural agrarian communities where rice growing is a major economic activity, (c) being socially-disadvantaged in terms of having lower average income compared to the provincial average value and unavailability of a standardized water irrigation system provided by Provincial Waterworks Authority (PWA), (d) using the groundwater from the community well used for village water supply for drinking and cooking with the improper treatment of fluoride in the water (i.e., boiling), and (e) being targeted areas of the local public health office’s initiative to establish a dental fluorosis surveillance system as a result of reports of dental fluorosis. This study initially aimed to include all 12 primary schools located in the 5 subdistricts. Only one school declined to serve as a research site. Finally, 11 schools provided permission and participated in this study. Eligible criteria for participants in this study were: (1) the first and second-grade students of the schools that provided permission and participated in this study; (2) have resided within the five subdistricts since birth; (3) their caregivers provided written consent for the participation in this research and (4) were present at schools on the day of an oral examination. After assessing their eligibility, all the first and second-grade students in all 11 schools were eligible and included, and none of them declined to participate.

### Study size

The study size was estimated according to the two research objectives. Firstly, to measure dental fluorosis prevalence, a study size of 203 individuals was estimated according to the method described by Daniel [29] to have a 95% confidence level and precision of 5% [30]. The value of the expected prevalence of dental fluorosis in the calculation was obtained from the reported 15.6% in a previous study in Thailand [20]. Secondly, to evaluate the association between fluoride in groundwater and dental fluorosis given unknown exposure proportions in the anticipated cases and controls in this community, the study size was estimated according to the method described in a previous case–control study conducted in a fluoridated community [31]. The study size of 34 cases and 34 controls were estimated to have a 95% confidence level, 80% statistical power, 5% exposure among controls, and an estimated risk ratio of 5.4 [31].

### Outcome measurement

In the first phase of the cross-sectional survey, the outcome of dental fluorosis was evaluated as a part of routine school-based oral health check-ups in all students. An authorized public health officer from the Nakhon Pathom Provincial Public Health Office, who has been in charge of the province's school oral health program and also served as an author (AS), collaborated with local public health officers in Bang Lan District to inform school principals of the dental check-up program and to obtain permission. Teachers of grade 1 and 2 students were informed of the program and requested to distribute informed consent forms to caregivers of their students. The public health officers scheduled an examination date with the schools after obtaining the signed informed consent forms. On the examination date, teachers brought students to the organized inspection area, facilitated and monitored the examination. The oral examination protocol was based on the standardized method of the National Oral Health Survey [32]. Dental fluorosis was examined by only one authorized dentist from Nakhon Pathom Provincial Public Health Office, who was also an author (PN). Intra-examiner calibration was carried during training for oral health examination and record of data for the National Oral Health Survey at Nakhon Pathom Provincial Public Health Office. Using Kappa statistics, intra-examiner consistency was assessed, and the result achieved was 0.9, indicating nearly perfect agreement [33]. Visual inspection on anterior teeth was a method of examination as recommended by the World Health Organization (WHO) for a practical reason [33]. Dean’s index (1942) was applied to classify the severity of dental fluorosis. Six levels of the classification include normal, questionable, very mild, mild, moderate, and severe [34]. From the survey result, children with very mild to severe fluorosis were regarded as ‘cases’ while their counterparts were ‘controls’ for a subsequent case–control study.

### Exposure measurement

In the case–control study, the exposure and behavioral factors of each child were traced backward until birth. The major exposure was fluoride concentration in groundwater used for household water supply. Since the Bureau of Dental Health, Ministry of Public Health, Thailand, has designated Bang Len District as an area under surveillance for dental fluorosis since 1999, water samples have been collected annually from drinking water sources (i.e., artesian wells, village water supplies) that covered all addresses of residents to be examined for fluoride content at the Bureau of Dental Health’s lab using ion-selective electrode technique. Fluoride concentration has been recorded correspondingly to the location and the source of water sampled. The database of fluoride concentration was then shared with the provincial public health office. For this study, annual records of fluoride concentrations in the groundwater used for the household water supply corresponding to the residence of each child from 2008 to 2015 were retrieved from the database at Nakhon Pathom Provincial Public Health Office. These records were verified by the public health officer who has routinely managed the database (AS).

Other explanatory variables included child’s sex and age, caregiver’s education, family income per month, breastfeeding, brushing frequency before and after 2 years old, toothpaste type, toothpaste size, and fluoride supplement. A child’s sex was classified as either male or female, and the age was from birth until 2015—stated in years. The family income per month was an estimated total monthly income of all family members expressed in Thai Bahts. The family income per month was dichotomously categorized during analysis using the average family expenditure per month in 2015 of 26,025 Thai Bahts (THB) in Nakhon Pathom. We classified sufficient income (≥ cut-off value) versus insufficient (< cut-off value) for family income. Breastfeeding was a dichotomous variable (yes/no) indicating whether the child had been breastfed for at least 6 months. Tooth brushing frequency was ascertained for the periods before and after 2 years old based on the difference in the child’s capability of tooth brushing [6, 35, 36]. Toothpaste size was dichotomously categorized into pea-sized and larger than pea-sized [6]. These explanatory variables were ascertained using an interview questionnaire (Additional file 1). The face-to-face interview with the caregivers was undertaken through the existing community public health network. The district public health officers trained village health volunteers on how to conduct face-to-face interviews for data collection and provided lists of students to be contacted in their villages. Each village health volunteer contacted caregivers of the assigned students to inform them about this research project, related procedures, and anticipated use of data they provided for subsequent research and local health system development. After informed consent was obtained, the volunteer interviewed the caregiver and recorded information in the paper questionnaire.

### Data management and statistical analysis

The dependent variable for this study was the prevalence of dental fluorosis in children. The prevalence was calculated by the number of children having very mild to severe dental fluorosis divided by the total number of children surveyed [37]. The main exposure was fluoride concentration in groundwater used for household water supply. For each child, available measures of groundwater fluoride concentrations in the drinking water source supplying the child’s residence were time-averaged over the period of birth to the survey. The time-averaged fluoride concentration was then categorized into three levels including < 0.7, 0.7–1.49, and ≥ 1.5 ppm respectively. These cut-off points were based on the locally recommended fluoride concentration in drinking water which is less than 0.7 ppm [20] and the WHO’s recommended fluoride concentration which is less than 1.5 ppm [7]. Other explanatory variables which were regarded as potential confounders in the analysis phase included the child’s demographic factors (age and sex), caregiver factors (education and family income), history of breastfeeding, fluoride supplementation, and children’s oral health behaviors (tooth brushing frequency, fluoride toothpaste use, and toothpaste size).

**Fig. 1:**
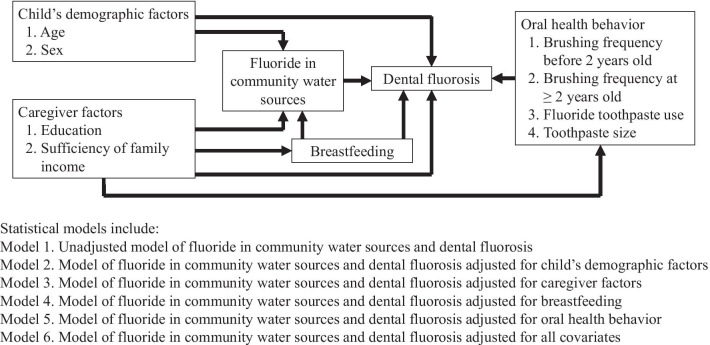
Directed acyclic graph of the analysis

Characteristics of children and caregivers were summarized using descriptive statistics. The exact probability test was applied to test for differences in proportions [38]. A Wilcoxon-type test for trend was applied to examine the trend of dental fluorosis occurrence across ordered levels of fluoride concentration [39]. Since the outcome of dental fluorosis was expected to be common especially in the fluoride endemic areas in Thailand [25], Poisson regression with robust standard errors was employed to estimate the effect measure of dental fluorosis prevalence ratio (PR) [40]. The technique was applied to avoid overestimating the odds ratio when the outcome occurrence is common [40–42]. Univariable analysis was conducted to evaluate the crude association between each explanatory variable and dental fluorosis. The univariable analysis of the main exposure of fluoride in village water sources and dental fluorosis served as the crude model (Model 1) providing an unadjusted prevalence ratio. Multivariable analysis was undertaken in sequence according to the conceptual model depicted by a directed acyclic graph (Fig. 1). Model 2 estimated an effect of the main exposure on dental fluorosis occurrence adjusted for the child’s demographic factors. Model 3 included the main exposure adjusted for caregiver factors. The effect of breastfeeding was adjusted in Model 4. Oral health behaviors including tooth brushing frequency and toothpaste use were controlled for their effects in Model 5. Model 6 included the main exposure adjusted for all covariates. The magnitude of confounding by the covariates could be quantified by the percentage difference between the crude and adjusted prevalence ratios. The formula for this calculation was (PR_crude_–PR_adjusted_)/PR_adjusted_. Regression post-estimation analysis of variance inflation factors for independent variables was also undertaken to evaluate the presence of multicollinearity [43].

## Results

There were 289 children whose ages ranged from 6 to 10 years old in this study. The median family income per month of these children was only 9000 Thai Bahts-or approximately 300 US dollars per month–which was much lower than the average family expenditure per month of 26,025 Thai Bahts in the whole Bang Len District of which these 5 subdistricts were a subset. Only 4% of the children were from families with a monthly family income above the average family expenditure value for the district. The majority of their caregivers had no schooling. A high breastfeeding proportion was reported by the caregivers. The mean of the time-averaged fluoride concentrations in the drinking water of the study area was 2.4 ppm with a maximum of 9.4 ppm (Table 1).

**Table 1:** Characteristics and dental fluorosis among children in Bang Len District, Nakhon Pathom, Thailand, 2015

Characteristic	Total n (%)^†^	Dental fluorosis status				
		Normal n (%)^‡^	Questionable n (%)	Very mild n (%)	Mild n (%)	Moderate n (%)
Overall	289	118 (40.8)	14 (4.9)	117 (40.5)	27 (9.3)	13 (4.5)
Study area						
Sai Ngam	65 (22.5)	26 (40.0)	6 (9.2)	20 (30.8)	8 (12.3)	5 (7.7)
Bang Sai Pa	40 (13.8)	23 (57.5)	0	14 (35.0)	2 (5.0)	1 (2.5)
Hin Mun	67 (23.2)	18 (26.8)	6 (9.0)	35 (52.2)	7 (10.5)	1 (1.5)
Bang Luang	106 (36.7)	41 (38.7)	2 (1.9)	47 (44.3)	10 (9.4)	6 (5.7)
Nin Phet	11 (3.8)	10 (90.9)	0	1 (9.1)	0	0
Sex						
Female	136 (47.1)	56 (41.2)	6 (4.4)	53 (38.9)	13 (9.6)	8 (5.9)
Male	153 (52.9)	62 (40.5)	8 (5.2)	64 (41.8)	14 (9.2)	5 (3.3)
Age (year)						
Mean ± SD	7.9 ± 0.8	7.8 ± 0.8	7.9 ± 0.8	7.9 ± 0.8	8.0 ± 0.6	8.2 ± 0.9
6	1 (0.4)	1 (100)	0	0	0	0
7	98 (33.9)	45 (45.9)	5 (5.1)	38 (38.8)	6 (6.1)	4 (4.1)
8	132 (45.6)	50 (37.9)	6 (4.6)	57 (43.1)	16 (12.1)	3 (2.3)
9	51 (17.7)	18 (35.3)	3 (5.9)	19 (37.2)	5 (9.8)	6 (11.8)
10	7 (2.4)	4 (57.1)	0	3 (42.9)	0	0
Caregiver’s education (n = 191)						
No schooling	90 (47.1)	32 (35.6)	3 (3.3)	39 (43.3)	10 (11.1)	6 (6.7)
Primary school	50 (26.2)	19 (38.0)	2 (4.0)	26 (52.0)	2 (4.0)	1 (2.0)
Secondary school	43 (22.5)	18 (41.8)	2 (4.7)	14 (32.6)	8 (18.6)	1 (2.3)
Vocational college	7 (3.7)	2 (28.6)	0	3 (42.8)	0	2 (28.6)
Undergraduate	1 (0.5)	0	1 (100.0)	0	0	0
Family income per month [in THB, 30 THB ≈ 1 USD]						
Respondent (n)	201	73	10	89	20	9
Median	9000	9000	9500	9000	10,000	9000
IQR	6000	5000	5000	4000	11,550	8000
Minimum	700	700	5000	1000	1500	3500
Maximum	50,000	30,000	50,000	50,000	30,000	40,000
Sufficiency of family income per month [≥ 26,025 THB]*						
Sufficient	8 (4.0)	1 (12.5)	1 (12.5)	3 (37.5)	2 (25.0)	1 (12.5)
Insufficient	193 (96.0)	72 (37.3)	9 (4.7)	86 (44.5)	18 (9.3)	8 (4.2)
Breastfeeding (n = 212)						
Yes	188 (88.7)	72 (38.3)	9 (4.8)	82 (43.6)	16 (8.5)	9 (4.8)
No	24 (11.3)	3 (12.5)	1 (4.2)	12 (50.0)	6 (25.0)	2 (8.3)
Brushing frequency before 2 years old (n = 215)						
No brushing	22 (10.2)	10 (45.4)	1 (4.6)	7 (31.8)	3 (13.6)	1 (4.6)
Once a day	120 (55.8)	34 (28.3)	8 (6.7)	60 (50.0)	14 (11.7)	4 (3.3)
Twice a day	65 (30.2)	31 (47.6)	1 (1.5)	25 (38.5)	4 (6.2)	4 (6.2)
> 2 times a day	8 (3.8)	1 (12.5)	1 (12.5)	5 (62.5)	0	1 (12.5)
Brushing frequency at ≥ 2 years old (n = 224)						
Once a day	63 (28.1)	24 (38.1)	4 (6.4)	26 (41.2)	7 (11.1)	2 (3.2)
Twice a day	129 (57.6)	46 (35.7)	8 (6.2)	56 (43.4)	11 (8.5)	8 (6.2)
> 2 times a day	32 (14.3)	10 (31.3)	0	16 (50.0)	5 (15.6)	1 (3.1)
Toothpaste type (n = 211)						
Non-fluoride	12 (5.7)	4 (33.3)	0	5 (41.7)	1 (8.3)	2 (16.7)
Fluoride	199 (94.3)	72 (36.2)	10 (5.0)	88 (44.2)	20 (10.1)	9 (4.5)
Toothpaste size (n = 220)						
Pea-sized	107 (48.6)	44 (41.1)	9 (8.4)	41 (38.3)	10 (9.4)	3 (2.8)
> Pea-sized	113 (51.4)	36 (31.8)	3 (2.7)	54 (47.8)	13 (11.5)	7 (6.2)
Fluoride supplement (n = 221)						
No	160 (72.4)	56 (35.0)	9 (5.6)	74 (46.2)	14 (8.8)	7 (4.4)
Yes	61 (27.6)	25 (41.0)	3 (4.9)	21 (34.4)	7 (11.5)	5 (8.2)
Time-averaged fluoride concentration (ppm)**						
Mean ± SD	2.4 ± 2.1	2.0 ± 1.6	1.7 ± 0.6	2.8 ± 2.2	2.8 ± 2.3	4.1 ± 3.5
Median (IQR)	1.9 (0.9)	1.6 (1.1)	1.7 (0.6)	2.0 (1.4)	2.1 (1.4)	2.0 (7.1)
Minimum	0.4	0.4	0.6	0.4	1.1	1.2
Maximum	9.4	9.4	3.0	9.4	9.4	9.4
< 0.7	30 (10.4)	22 (73.3)	1 (3.4)	7 (23.3)	0	0
0.7–1.49	61 (21.1)	33 (54.1)	5 (8.2)	14 (23.0)	6 (9.8)	3 (4.9)
≥ 1.5	198 (68.5)	63 (31.8)	8 (4.1)	96 (48.4)	21 (10.6)	10 (5.1)

SD, standard deviation; IQR, interquartile range; THB, Thai Baht; USD, US Dollar; ppm, parts per million

^†^Column percentage; ^‡^Row percentage

^*^Average family expenditure per month in 2015, Nakhon Pathom, Thailand

^**^For each child, available measures of groundwater fluoride concentrations in drinking water sources supplying the child’s residence were time-averaged over the period of birth to the survey

According to Table 1, there were 157 children (54.3%) having dental fluorosis with severity ranging from very mild to moderate level. None was found to have severe dental fluorosis. Considerably higher dental fluorosis prevalence was determined among children who used more than a pea-sized amount of toothpaste (65.5%) compared to the 50.5% prevalence in their counterparts (Exact probability test; *P* = 0.029). The prevalence among children who were not breastfed (83.3%) was substantially greater than the 56.9% prevalence among those who were breastfed (Exact probability test; *P* = 0.014). A significant positive trend between the time-averaged fluoride concentrations in drinking water and dental fluorosis prevalence was determined (Test for trend; *P* < 0.001). The dental fluorosis prevalences were 23.3%, 37.7%, and 64.1% corresponding to the levels of the time-averaged fluoride concentrations in drinking water of < 0.7, 0.7–1.49, and ≥ 1.5 ppm respectively (Exact probability test; *P* < 0.001). Moreover, the severity of dental fluorosis in all 7 cases whose time-averaged fluoride concentrations were < 0.7 ppm was limited to the very mild level only. Higher prevalences of dental fluorosis with mild (10.6%) and moderate (5.1%) severity levels were also observed among children whose time-averaged fluoride concentrations were ≥ 1.5 ppm compared to the dental fluorosis prevalences with mild (9.8%) and moderate (4.9%) severity in the group with the time-averaged fluoride concentrations of 0.7–1.49 ppm. Note that the exact probability test and the chi-square test for trend were applicable only for some variables. The test results were thus explained here and not in Table 1.

**Fig. 2:**
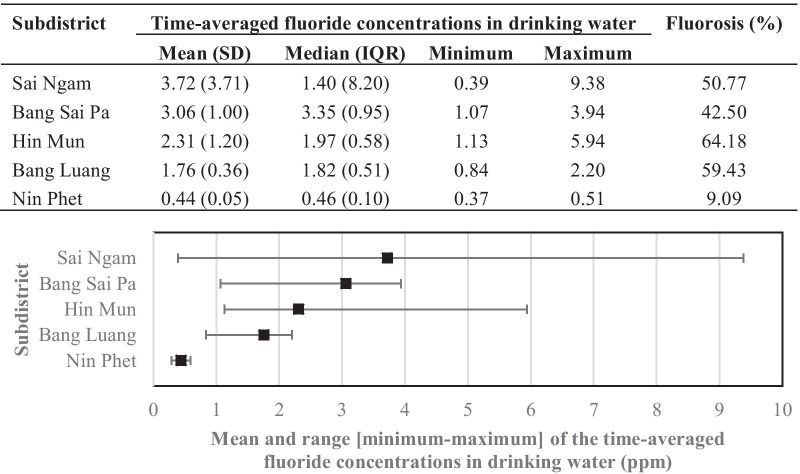
Mean and range of time-averaged fluoride concentrations by subdistrict

The mean and range of the time-averaged fluoride concentrations and corresponding prevalence of dental fluorosis in the 5 subdistricts were summarized in Fig. 2. The highest mean (3.72 ppm) with the widest range of fluoride concentrations (0.39–9.38 ppm) was observed in Sai Ngam subdistrict. In contrast, the lowest mean (0.44 ppm) with the narrowest range (0.37–0.51 ppm) of fluoride concentrations was obtained from 11 children living in Nin Phet subdistrict. Hin Mun subdistrict had the highest prevalence of dental fluorosis while having neither the greatest mean nor median fluoride concentrations. Nonetheless, almost all of the fluoride concentrations in children’s household water sources in Hin Mun subdistrict, ranging from 1.13 to 5.94 ppm, were higher than the WHO's recommended fluoride level of less than 1.5 ppm.

The findings of the univariable analysis using Poisson regression with robust standard errors to estimate crude prevalence ratio are presented in Table 2. Prevalence of dental fluorosis among the children with the time-averaged fluoride concentrations in drinking water of ≥ 1.5 ppm was 2.75 times the prevalence among those with the fluoride concentrations of < 0.7 ppm (95% CI; 1.42, 5.31). A significantly higher prevalence of dental fluorosis was determined among children who were not breastfed compared to that of their counterparts. The prevalence of dental fluorosis among children using more than a pea-sized amount of toothpaste was 1.3 times the prevalence in the group using a pea-sized amount of toothpaste (95% CI; 1.03, 1.63).

**Table 2:** Univariable analysis of crude association between dental fluorosis and each explanatory variable

Variable	Cases n (%)^†^	Controls n (%)^†^	Univariable model^‡^		
			PR	95% CI	*P*-value
Total	132	157			
*Main exposure variable*					
Time-averaged fluoride concentration (ppm)*					
< 0.7	7 (23.3)	23 (76.7)		Reference	
0.7–1.49	23 (37.7)	38 (62.3)	1.62	0.78, 3.34	0.195
≥ 1.5	127 (64.1)	71 (35.9)	2.75	1.42, 5.31	0.003
*Other explanatory variables*					
Sex					
Female	74 (54.4)	62 (45.6)		Reference	
Male	83 (54.3)	70 (45.7)	1.0	0.81, 1.23	0.978
Age (year)					
< 8	48 (48.5)	51 (51.5)		Reference	
≥ 8	109 (57.4)	81 (42.6)	1.18	0.93, 1.50	0.165
Caregiver’s education					
≥ Secondary school	28 (54.9)	23 (45.1)		Reference	
< Secondary school	84 (60.0)	56 (40.0)	1.09	0.82, 1.45	0.540
Sufficiency of family income per month [≥ 26,025 THB]**					
Sufficient	6 (75.0)	2 (25.0)		Reference	
Insufficient	112 (58.0)	81 (42.0)	0.77	0.51, 1.18	0.230
Breastfeeding					
Yes	107 (56.9)	81 (43.1)		Reference	
No	20 (83.3)	4 (16.7)	1.46	1.18, 1.82	0.001
Brushing frequency before 2 years old					
No brushing	11 (50.0)	11 (50.0)		Reference	
Once a day	78 (65.0)	42 (35.0)	1.30	0.84, 2.02	0.241
≥ 2 times a day	39 (53.4)	34 (46.6)	1.07	0.67, 1.71	0.783
Brushing frequency at ≥ 2 years old					
Once a day	35 (55.6)	28 (44.4)		Reference	
≥ 2 times a day	97 (60.2)	64 (39.8)	1.08	0.84, 1.40	0.532
Fluoride toothpaste use					
No	8 (66.7)	4 (33.3)		Reference	
Yes	117 (58.8)	82 (41.2)	0.88	0.58, 1.34	0.555
Toothpaste size					
Pea-sized	54 (50.5)	53 (49.5)		Reference	
> Pea-sized	74 (65.5)	39 (34.5)	1.30	1.03, 1.63	0.027
Fluoride supplement^§^					
No	95 (59.4)	65 (40.6)		Reference	
Yes	33 (54.1)	28 (45.9)	0.91	0.70, 1.19	0.491

PR, Prevalence ratio

^†^Row percentage is presented to comply with analysis of prevalence ratio

^‡^Univariable model estimated by Poisson regression with robust standard error

^*^For each child, available measures of groundwater fluoride concentrations in drinking water sources supplying the child’s residence were time-averaged over the period of birth to the survey

^**^Average family expenditure per month in 2015, Nakhon Pathom, Thailand

^§^Fluoride supplement was not further included in the multivariable model due to the collinearity problem

The multivariable analysis of dental fluorosis prevalence using Poisson regression with robust standard errors adjusting for covariates is presented in Table 3. The unadjusted analysis (Model 1) provides crude estimates of prevalence ratio comparing the two index categories–the time-averaged fluoride concentrations in drinking water of 0.7–1.49 and ≥ 1.5 ppm–to the same referent category of the time-averaged fluoride concentrations in drinking water of < 0.7 ppm. Comparing the prevalence of dental fluorosis between the first index category of fluoride concentrations of 0.7–1.49 ppm and the referent category, the adjusted prevalence ratios in Model 2 and 3 remained similar to the crude estimate in Model 1. In contrast, the adjusted prevalence ratios in Models 4 and 5 were considerably greater than the crude estimate. However, none of these crude and adjusted prevalence ratios were statistically significant. Comparing between the second index category of the time-averaged fluoride concentrations of ≥ 1.5 ppm and the reference; the adjusted prevalence ratio in Model 2 was 2.78 (*P* = 0.003) which was slightly greater than the crude prevalence ratio of 2.75 in Model 1. In Models 3–5, the adjusted prevalence ratios further increased to 2.81, 5.30 and 6.46 respectively. Nonetheless, statistical significance was no longer maintained in these three subsequent models. After adjusting for all covariates (Model 6), the adjusted prevalence ratios in both index categories were close to their corresponding crude estimates (Model 1). The adjusted prevalence ratio comparing the first index category (0.7–1.49 ppm) to the referent category slightly increased to 1.64 with a wider confidence interval (95% CI; 0.24, 11.24). The adjusted prevalence ratio comparing the second index category (≥ 1.5 ppm) to the referent category increased to 2.85, though not statistically significant and a considerably wider confidence interval was obtained (95% CI; 0.44, 18.52). The magnitude of confounding could be quantified by calculating the percentage difference between the crude estimate (Model 1) and the adjusted estimate (Model 6) of prevalence ratio for each index category. For the first index category of fluoride concentrations of 0.7–1.49 ppm, the magnitude of confounding was − 1.2%, which was calculated by [(1.62–1.64)/1.64] × 100%. For the second index category of fluoride concentrations of ≥ 1.5 ppm, the magnitude of confounding was − 3.5%, which was calculated by [(2.75–2.85)/2.85] × 100%.

**Table 3:** Multivariable models for dental fluorosis

Variable	Model 1, crude model (unadjusted)			Model 2, adjusted for child’s demographic factors			Model 3, adjusted for caregiver factors			Model 4, adjusted for breastfeeding			Model 5, adjusted for oral health behaviors			Model 6, adjusted for all covariates		
	PR [95% CI]	*P*-value		PR [95% CI]	*P*-value		PR [95% CI]	*P*-value		PR [95% CI]	*P*-value		PR [95% CI]	*P*-value		PR [95% CI]	*P*-value	
*Main exposure variable*																		
Time-averaged fluoride concentration (ppm)*																		
< 0.7	Reference			Reference			Reference			Reference			Reference			Reference		
0.7–1.49	1.62 [0.78, 3.34]	0.195		1.62 [0.79, 3.32]	0.190		1.61 [0.28, 9.21]	0.592		3.08 [0.47, 20.04]	0.238		3.44 [0.48, 24.62]	0.218		1.64 [0.24, 11.24]	0.615	
≥ 1.5	2.75 [1.42, 5.31]	0.003		2.78 [1.45, 5.32]	0.002		2.81 [0.51, 15.51]	0.235		5.30 [0.84, 33.45]	0.076		6.46 [0.94, 44.48]	0.058		2.85 [0.44, 18.52]	0.273	
*Child’s demographic factors*																		
Sex																		
Female				Reference												Reference		
Male				1.06 [0.86, 1.29]	0.596											1.07 [0.83, 1.37]	0.615	
Age (year)																		
< 8				Reference												Reference		
≥ 8				1.19 [0.95, 1.50]	0.125											1.00 [0.75, 1.35]	0.976	
*Caregiver factors*																		
Caregiver’s education																		
≥ Secondary school							Reference									Reference		
< Secondary school							1.25 [0.92, 1.69]	0.149								1.24 [0.91, 1.69]	0.178	
Sufficiency of family income																		
Sufficient							Reference									Reference		
Insufficient							0.84 [0.55, 1.30]	0.442								0.81 [0.51, 1.29]	0.378	
*Breastfeeding factor*																		
Breastfeeding																		
Yes										Reference						Reference		
No										1.28 [1.04, 1.57]	0.019					1.31 [0.97, 1.77]	0.078	
*Oral health behaviors*																		
Brushing frequency before 2 years old																		
No brushing													Reference			Reference		
Once a day													1.29 [0.80, 2.08]	0.303		1.24 [0.76, 2.03]	0.397	
≥ 2 times a day													0.94 [0.55, 1.59]	0.805		0.95 [0.55, 1.65]	0.854	
Brushing frequency at ≥ 2 years old																		
Once a day													Reference			Reference		
≥ 2 times a day													1.18 [0.89, 1.56]	0.253		1.16 [0.83, 1.63]	0.378	
Fluoride toothpaste use																		
No													Reference			Reference		
Yes													1.06 [0.68, 1.65]	0.797		1.23 [0.46, 3.28]	0.685	
Toothpaste size																		
Pea-sized													Reference			Reference		
> Pea-sized													1.20 [0.96, 1.50]	0.108		1.24 [0.94, 1.63]	0.130	

Multivariable models were estimated by Poisson regression with robust standard errors

PR, Prevalence ratio

*For each child, available measures of groundwater fluoride concentrations in drinking water sources supplying the child’s residence were time-averaged over the period of birth to the survey

## Discussion

The prevalence of dental fluorosis in children living in fluoride endemic locations has been shown to vary substantially across studies conducted with similar rural study settings. Dental fluorosis prevalence ranged from 28% in the Ethiopian Rift Valley to 98% in Oaxaca, Mexico [7, 44]. In epidemiological surveys from China, India, and Indonesia; the overall prevalence of dental fluorosis regardless of severity levels were 38.2%, 69.4% and 96.0%, respectively [45–47]. In Thailand, the overall prevalence ranged from 5% (very mild to severe dental fluorosis) in Panomsarakham District, Chachoengsao Province [20], to 70.9% (Thylstrup and Fejerskov Index, level 1–4) in Chiang Mai Province [25]. The finding of 54.3% prevalence in this study was considered relatively high given the global context and especially when compared to the 5% prevalence in Chachoengsao Province which was only 150 km east of the current study site. The substantially lower prevalence in Chachoengsao Province could be related to the high proportion of 87.5% of children living in areas with water supplies containing fluoride less than 0.7 ppm. In this study, however, 89.6% of the children had household water sources with fluoride contents of ≥ 7 ppm. This large disparity of the prevalence in these two comparable settings in Thailand implied the crucial effect of endemic fluoride on dental fluorosis occurrence at the population level.

In addition, this plausible effect of natural fluoride in groundwater use for household consumption on the overall and severity-specific prevalence in this study was also comparable with the ones observed in Birigui, SP, Brazil. In that study with a socio-environmental setting and methods of exposure and outcome measurement resembled the current study; the overall prevalence was 58.9% and severity-specific prevalence values were 44.4% for very mild, 11.9% for mild, 2.4% for moderate, and 0.2% for severe dental fluorosis [37]. This evidence further demonstrated the quality of consistency in the effect of endemic fluoride on the prevalence of dental fluorosis which was observed in different groups of children, at different times, and in different places [48, 49].

The biological gradient between fluoride concentrations in groundwater used for household water supply and dental fluorosis occurrence in children was suggested by the unidirectional positive relationship of these attributes in the current study. The finding of 23.3% prevalence with only the very mild dental fluorosis among children with time-averaged fluoride concentrations of < 0.7 ppm (the referent category) was evidence that reassured the safety of this recommended optimal fluoride level in this setting and the others [20, 37]. When the fluoride concentrations increased to the range of 0.7–1.49 ppm (index category 1), the prevalence among children in this group also increased to 37.7%, with the additional higher levels of mild and moderate severity. Although the fluoride concentrations in this range did not surpass the WHO's recommended limit of 1.5 ppm [7], the results of this study were concerning as the prevalence exceeded one-third of the children and 14.7% of the severity was beyond the very mild level. In the extreme group with the fluoride ≥ 1.5 ppm (index category 2), the prevalence further rose to 64.1% or approximately 2.8 times the prevalence of those in the reference group. The severity beyond the very mild level also grew to 15.7%. This finding of the biological gradient suggested the rational use of fluoride concentrations in household water sources as an indicator for the possible occurrence of dental fluorosis and related severity.

Multivariable regression models constructed according to the DAGs, which displayed various assumptions regarding the main association between the time-average fluoride concentrations and dental fluorosis given a different set of socio-behavioral determinants being simultaneously considered in each model, provided insight into the etiologic pattern of dental fluorosis in this fluoride endemic setting (Table 3). When the measure of association between fluoride concentrations and dental fluorosis was compared before and after adjusting for the child’s demographic factors (sex and age) and caregiver’s factors (education and sufficiency of family income), the difference was negligible, indicating the minimal confounding caused by these factors. Lack of association between child’s sex and dental fluorosis; indicated by crude PR of 1.0 in Table 2 and adjusted PRs of 1.06 (Model 2) and 1.07 (Model 6) in Table 3, would explain its trifling influence on the main association between fluoride concentrations and dental fluorosis. The insignificant difference in dental fluorosis occurrence between Thai male and female children, aged 8–10 years old, and the lack of association between child’s sex and dental fluorosis-indicating by statistically non-significant crude and adjusted odds ratios of 1.2 and 0.9-were consistently observed in a previous study [50]. Regarding the child’s age, several studies conducted in fluoride endemic areas have contrastively demonstrated a positive linear association between age and dental fluorosis prevalence [12]. The association in those studies might be attributable to the more diverse age categories ranging from 3 to 18 years of age among the study participants and the more visible dental fluorosis in children aged over 10 years old compared to the ones below the age of 8 [12]. Although children cared for by the caregivers with less than a secondary school education had a 5.1% higher prevalence of dental fluorosis, the negative association between lower caregiver’s education and higher dental fluorosis was not supported by the findings of crude and adjusted PRs close to 1 and statistical non-significance. The prior investigation in children living in Bangkok consistently revealed that dental fluorosis prevalence among children having caregivers with education higher than bachelor’s degree was not significantly lower than the prevalence among those having caregivers with lower levels of education (odds ratio, 0.85; 95% CI, 0.54–1.33) [50]. The lack of association between caregiver’s education and child dental fluorosis might be attributable to the fact that knowledge regarding dental fluorosis and its prevention has never been included in general education in Thailand. This suggested the need for a community-based educational effort to improve literacy regarding dental fluorosis prevention among caregivers of children with developing dentition. In this study, insufficient family income was hypothesized to be associated with higher dental fluorosis prevalence due to an assumption that children in poorer families might be more likely to expose to fluoride in groundwater than their counterparts whose families might be able to afford alternative water sources for drinking such as bottled water. The results, however, did not support this hypothesis. This might be because all the 8 children from families with sufficient income had a household water supply with fluoride concentrations of ≥ 1.5 ppm. Another study in Bangkok unveiled a significant relationship between the higher class of family income and greater dental fluorosis prevalence (odds ratio, 1.77; 95% CI, 1.10–2.86) [50]. It was suggested that families with greater income might be more capable of purchasing fluoride products and the children in these families would be more likely to have increased exposure to fluoride [50, 51]. Therefore, the effect of family income on dental fluorosis may vary by different contexts of studies.

The association between fluoride concentrations and dental fluorosis was heightened after adjusting for the effect of breastfeeding and children’s oral health behaviors (Table 3). The dental fluorosis PR comparing the fluoride concentrations of 0.7–1.49 ppm (index category 1) to < 0.7 ppm (the referent category) considerably increased from 1.62 (Model 1) to 3.08 (Model 4) and 3.44 (Model 5), indicating confounding magnitudes of − 47.4% by breastfeeding and − 52.9% by children’s oral health behaviors. The fluorosis PR comparing the concentrations of ≥ 1.5 ppm (index category 2) to the referent category also greatly increased from 2.75 (Model 1) to 5.30 (Model 4) and 6.46 (Model 5), showing confounding magnitudes of − 48.1% by breastfeeding and − 57.4% by children’s oral health behaviors. The large confounding magnitudes created by these factors highlighted their influential role in the etiologic mechanism of dental fluorosis in the fluoride-endemic environment, accentuating the need to consider these factors when measuring the exposure-outcome association between natural fluoride in water used for consumption and dental fluorosis in settings similar to the current ones. Regarding the role of breastfeeding, a prolonged period of breastfeeding has been demonstrated to protect against dental fluorosis [52–55]. Breast milk was shown to have only a trace amount of fluoride regardless of the quantity of fluoride consumed by mothers [56]. Breastfeeding during the first two years of life, which aligns with the active period of enamel formation, thus appears to prevent dental fluorosis [54]. In the current study, breastfeeding might reduce the exposure to fluoride through the use of water containing natural fluoride to prepare powdered formula. Concerning the role of children’s oral health behaviors, early toothbrushing before 2 years old was shown to be strongly associated with mild-to-moderate fluorosis [6]. This habit was also common in this study, having 89.8% of children brushing once a day or more. Fluoride exposure could be heightened in this context as this habit coincided with 94.3% of children using fluoride toothpaste and 51.4% of children using more than pea-sized toothpaste amounts. Ingesting toothpaste during the period of developing enamel could lead to dental fluorosis [57] and this would explain the collective influence of children’s oral health behaviors on dental fluorosis in this setting. Ultimately, the main association between fluoride concentrations and dental fluorosis attenuated after taking full control of all covariates (Model 6), ruling out alternative explanations given information of all other covariates for the observed effect of higher classes of fluoride concentrations (index category 1 and 2) on increased dental fluorosis prevalence beyond the ones of the referent category. By comparing the prevalence ratios for both index categories of fluoride concentrations in Model 6 to Model 1, the adjusted estimates differed only slightly from their corresponding crude estimates and the confounding magnitudes of − 1.2% and − 3.5% created by all covariates simultaneously considered in Model 6 were trivial. The limited confounding effect of all covariates allowed a conclusion to be drawn based on the crude estimates [58] that consumption of groundwater containing natural fluoride concentrations beyond 0.7 ppm increased the prevalence of dental fluorosis, particularly fluoride levels of 1.5 ppm or higher significantly increased the prevalence by 2.75 times compared to the ones of fluoride levels below 0.7 ppm.

DAGs have brought a new perspective on evaluating exposure-outcome associations by depicting plausible causal pathways that take into consideration the interplay of etiologic factors [17]. Unlike conventional univariable regression analysis, which evaluates the one-on-one statistical association between each independent variable and an outcome to determine which explanatory variables to be included in a multivariable model; DAGs depict the various roles of independent variables (i.e., main exposure, confounder, mediator) in the causal pathways that ease the selection of potential confounders and avoid adjusting for mediator variables [59]. Statistical adjustment by multivariable regression that adopts the list of significant explanatory variables from the aforementioned univariable analysis could even produce a biased or over-adjusted estimate if collider or intermediate variable is controlled [60]. Furthermore, a multivariable model that controls the effect of all extraneous variables simultaneously would not allow the measurement of confounding magnitude caused by each subset of extraneous variables, limiting knowledge of the relative influence of different extraneous variables on the association of interest. DAGs can serve as a conceptual framework for conducting multivariable analyses that sequentially include different sets of extraneous variables. This technique has been previously applied in dental research to elucidate a variation in etiologic patterns of early childhood caries [61, 62]. This study pioneered in applying the approach in dental fluorosis research and illustrating the need of taking into account the substantial confounding influence of breastfeeding and children’s oral health behavior on the association between natural fluoride in groundwater and dental fluorosis in children.

This study was limited in the use of an external outcome assessor due to the lack of trained dentists working at the local public health officials. To enable the oral examination by using a qualified dentist who served as a co-investigator of this study might also raise concern regarding bias in outcome assessment. Therefore, we validated the comparability of the dental fluorosis outcome in this study with previous records of routine dental fluorosis examinations in the same area that were archived at the provincial public health office. Recall bias might also be another concern since this study traced the past exposure status until the birth of each child. Nonetheless, caregivers of children with different fluorosis outcomes would not have differential attempts to recall since the fluorosis examination results were not disclosed until the end of the data collection phase. Recall errors from not being able to recall would also be possible. However, the exposure variables being recalled were relatively persistent (i.e., caregiver’s education) or habitual (i.e., breastfeeding) which would ease valid recall.

The strength of this study primarily lied in its study participants who could reflect the population-level dental fluorosis problem and pave the path for public health initiatives needed to address the condition in future generations of children in these fluoride endemic areas. The case–control study design along with the application of causal directed acyclic graphs not only allowed evaluation of the temporal association between natural fluoride in groundwater and dental fluorosis but also provided a plausible elucidation of the dental fluorosis’s etiologic pattern taken various socio-behavioral determinants into account. The evidence of high dental fluorosis prevalence necessitated multi-level public health initiatives to manage the problem. Children who were affected by dental fluorosis in the forms of physical damage or apparent stain on dental enamel should be provided with access to public dental care. Public communication to inform and educate the residents, especially the families of children at the ages of developing dentition, about dental fluorosis and its prevention should be carried out by local public health officers and village health volunteers. Fluoride mapping based on the annual records of fluoride concentrations in the groundwater used for household water supply should be utilized to identify water sources with ≥ 0.7 ppm fluoride which must be avoided for drinking and cooking. Engaging all community stakeholders to have shared accountability in developing a solution for safe water allocation to all residents should be implemented. School-based dental fluorosis surveillance should continue to monitor the situation of dental fluorosis and further provide information for pertinent public health actions.

## Conclusion

In fluoride endemic areas, groundwater containing natural fluoride utilized for household consumption resulted in high dental fluorosis prevalence, particularly in the groundwater with fluoride concentrations of ≥ 1.5 ppm.

## Supplementary Information

**Additional file 1**. Interview questionnaire.

## Data Availability

The datasets used and/or analysed during the current study are available from the corresponding author on reasonable request.

## Abbreviations

CI: Confidence interval
DAGs: Directed acyclic graphs
mg/L: Milligrams per liter
ppm: Parts per million
PR: Prevalence ratio
PR_crude_: Crude prevalence ratio
PR_adjusted_: Adjusted prevalence ratio
WHO: World Health Organization
