# Supplementary material for: Hydrogeogenic fluoride in groundwater and dental fluorosis in Thai agrarian communities: a prevalence survey and case–control study
Source: BMC Oral Health. 2021 Oct 22;21:545. doi: 10.1186/s12903-021-01902-8 (PMC8532340; doi:10.1186/s12903-021-01902-8)
Supplement: Supplementary file 1 — Additional file 1. Interview questionnaire. [file 12903_2021_1902_MOESM1_ESM.docx]

**Interview questionnaire**

(English translation)

Research ID: ______________

| **Question:** | **Variable:** | **Code:** |
| --- | --- | --- |
| 1. | Study area:   \| Sai Ngam \| \| --- \| \| Bang Sai Pa \| \| Hin Mun \| \| Bang Luang \| \| Nin Phet \| \|  \| | \| 1 \| \| --- \| \| 2 \| \| 3 \| \| 4 \| \| 5 \| |
| 2. | Caregiver’s education   \| No schooling \| \| --- \| \| Primary school \| \| Secondary school \| \| Vocational college \| \| Undergraduate \| \|  \| | \| 1 \| \| --- \| \| 2 \| \| 3 \| \| 4 \| \| 5 \| |
| 3. | The family income per month (Thai Bahts) | _____________________________ |
| 4. | Breastfeeding ≥ 6 months   \| Yes \| \| --- \| \| No \| | \| 1 \| \| --- \| \| 2 \| |
| 5. | Brushing frequency before child’s 2 years of age   \| No brushing \| \| --- \| \| Once a day \| \| Twice a day \| \| > 2 times a day \| \|  \| | \| 1 \| \| --- \| \| 2 \| \| 3 \| \| 4 \| |
| 6. | Brushing frequency after child’s 2  years of age   \| No brushing \| \| --- \| \| Once a day \| \| Twice a day \| \| > 2 times a day \| \|  \| | \| 1 \| \| --- \| \| 2 \| \| 3 \| \| 4 \| |
| 7. | Child’s toothpaste type  Brand of tooth paste: _______________  Having fluoride:   \| Yes \| \| --- \| \| No \| | \| 1 \| \| --- \| \| 2 \| |
| 8. | Child’s toothpaste size   \| Pea-sized \| \| --- \| \| > Pea-sized \| | \| 1 \| \| --- \| \| 2 \| |
| 9. | Fluoride supplement   \| Yes \| \| --- \| \| No \| | \| 1 \| \| --- \| \| 2 \| |
